# Bonding network and stability of clusters: the case study of Al_13_TM_4_ pseudo-tenfold surfaces

**DOI:** 10.1107/S2053273319000202

**Published:** 2019-02-28

**Authors:** Philippe Scheid, Corentin Chatelier, Julian Ledieu, Vincent Fournée, Émilie Gaudry

**Affiliations:** a Mines Nancy, Université de Lorraine, Campus Artem, Nancy, France; b Université de Lorraine, CNRS UMR7198, Institut Jean Lamour, F-54000 Nancy, France; c Synchrotron SOLEIL-CNRS, L’Orme des Merisiers, Saint-Aubin, BP48, 91192 Gif-sur-Yvette, France

**Keywords:** complex intermetallic compounds, surfaces, bonding, density functional theory

## Abstract

Clusters, *i.e.* polyhedral geometric entities, are widely used to describe the structure of complex intermetallic compounds. However, little is generally known about their physical significance. The atomic and electronic structures of the Al_13_TM_4_ complex intermetallic compounds (TM = Fe, Co, Ru, Rh) have been investigated using a wide range of *ab initio* tools in order to examine the influence of the chemical composition on the pertinence of the bulk structure description based on 3D clusters. In addition, since surface studies were found to be a relevant approach to address the question of cluster stability in complex phases, the interplay of the cluster substructure with the 2D surface is addressed in the case of the Al_13_Co_4_(100) and Al_13_Fe_4_(010) surfaces.

## Abstract 2

The physical significance of clusters in Al_13_TM_4_ compounds is invesitgated through *ab initio* methods based on density functional theory.

## 1. Introduction   

A large variety of intermetallic crystal structures are based on polyhedral entities, often called ‘clusters’, as first introduced by F. A. Cotton in the early 1960s to describe compounds containing metal–metal bonds (Cotton & Walton, 1982 ▸). This approach is very useful to represent the structures of complex intermetallic phases, like intermetallic clathrates, but also quasicrystals, their crystalline approximants and related phases (Steinhardt & Jeong, 1996 ▸; Suck *et al.*, 2002 ▸; Abe *et al.*, 2004 ▸). Typical clusters found in quasicrystals include the Mackay (Sugiyama *et al.*, 1998 ▸), Bergman (Bergman *et al.*, 1957 ▸) and Tsai clusters (Takakura *et al.*, 2001 ▸), and numerous polyhedral shapes are used to describe complex intermetallics.

**Figure 1:**
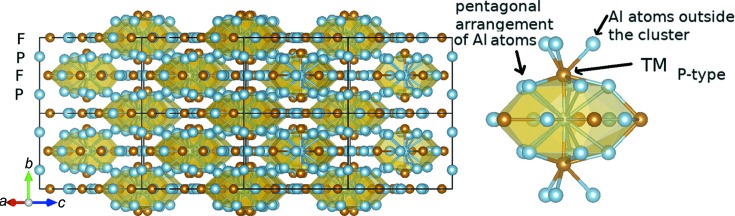
Left: Al_13_Fe_4_ bulk structure, highlighting (i) the description based on the stacking of atomic planes perpendicular to the [010] direction (F- and P-type planes) and (ii) the description based on the Henley-type clusters. Right: Henley-type cluster.

There is not usually a unique description of crystal structures in terms of structural building blocks. For example, a packing of pentagonal bipyramids was initially used by Henley to describe the Al_13_TM_4_ structure types (TM = Fe, Co, Ru, Rh, Fig. 1 ▸). Later, based on quantum chemistry calculations (Armbrüster *et al.*, 2011 ▸), these compounds were represented as columns of elongated clusters containing strong TM–Al–TM molecular groups, resembling the 3D ‘cage-compound’ structure of the intermetallic clathrates. Dong’s cluster+glue model, describing the structure by the [icosahedron](glue)_0,1_ formula containing all the key information on alloy chemistry (Dong *et al.*, 2007 ▸), uses two types of icosahedral clusters for Al_13_Fe_4_: Al_9_Fe_4_ and Fe_2_Al_11_ (Chen *et al.*, 2014 ▸). Finally, the 18 − *n* bonding scheme applied to Al_13_Os_4_ describes the structure by a stacking of Al_5_ square pyramids and fluorite-type columns (Miyazaki *et al.*, 2017 ▸).

The question of cluster stability in complex intermetallic compounds was reviewed a few years ago in the case of quasicrystals (Steurer, 2006 ▸). Several studies mention the influence of the intrinsic cluster structure on the bulk physical properties, such as their mechanical properties (Feuerbacher *et al.*, 2001 ▸; Messerschmidt *et al.*, 1999 ▸) or their electronic conductivity (Janot & de Boissieu, 1994 ▸; Janot, 1996 ▸; Trambly de Laissardière *et al.*, 1997 ▸, 2006 ▸; Fujiwara, 1989 ▸; Fujiwara *et al.*, 1993 ▸; Zijlstra & Bose, 2003 ▸; Trambly de Laissardière & Fujiwara, 1994 ▸; Trambly de Laissardière & Mayou, 1997 ▸; Dolinšek *et al.*, 2000 ▸; Stadnik *et al.*, 2001 ▸; Widmer *et al.*, 2006 ▸; Zijlstra & Janssen, 2000 ▸; Trambly de Laissardière, 2003 ▸, 2009 ▸). Molecular dynamics based modelling of crack and dislocation propagation in simple quasiperiodic model structures (Mikulla *et al.*, 1998 ▸; Rösch *et al.*, 2004 ▸, 2005 ▸; Rudhart *et al.*, 2004 ▸) also questioned the role of clusters. Surface studies were found to be a pertinent approach to gain some insight into the question of cluster stability in quasicrystals (Dubois & Belin-Ferré, 2011 ▸; McGrath *et al.*, 2010 ▸, and references therein). When prepared by sputtering and annealing cycles, quasicrystalline surfaces usually produce large, atomically flat terraces. The patterns observed by scanning tunnelling microscopy (STM) are attributed to signatures of the dissected clusters at the surface (Krajčí & Hafner, 2005 ▸). This then suggests that the clusters in icosahedral quasicrystals are not stable sub-units that maintain their shapes at the surface. Do these conclusions present a general character among complex phases? Recently, several surfaces of a cage compound – namely the type-I clathrate BaAuGe – were investigated by a combination of experimental and theoretical techniques. It was found that these surfaces preserve the cluster substructure, which is stabilized by a charge transfer mechanism (Anand *et al.*, 2018*a* ▸,*b* ▸).

**Figure 2:**
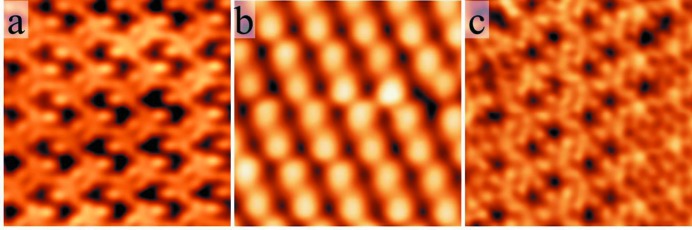
High-resolution STM images (6 × 6 nm). (*a*) o-Al_13_Co_4_ (*V*_*b*_ = −0.5 V), (*b*) Al_13_Fe_4_ (*V*_*b*_ = +1 V), (*c*) Al_13_Ru_4_ (*V*_*b*_ = −1.05 V).

It follows that different surface behaviours occur among complex phases. In the following, we focus on the Al_13_TM_4_ complex intermetallics (TM = Fe, Co, Ru, Rh), usually considered as four-layer decagonal quasicrystalline approximants, because they present a layered structure and pentagonal atomic arrangements (Goldman & Kelton, 1993 ▸). Experimentally, using single-crystal surfaces prepared by cycles of sputtering and annealing, significant differences were found among the considered pseudo-tenfold Al_13_TM_4_ (TM = Fe, Co, Ru) surface structures (Fournée *et al.*, 2012 ▸; Ledieu *et al.*, 2013 ▸, 2017 ▸; Shin *et al.*, 2011 ▸), although common features were also observed. For all considered systems, the surface composition measured from XPS (X-ray photoelectron spectroscopy) as a function of surface sensitivity indicated no sign of chemical segregation. A surface plane selection is observed for all Al_13_TM_4_ pseudo-tenfold surfaces, highlighted by STM measurements which identified consecutive terraces separated by a unique step height equal to half the lattice parameter. However, the absence of surface reconstruction is not observed systematically: the LEED (low-energy electron diffraction) patterns of the Al_13_Co_4_(100) and Al_13_Fe_4_(010) surfaces show rectangular and oblique surface unit cells, respectively, with dimensions consistent with the bulk values, while the surface of Al_13_Ru_4_(010) exhibits an atypical surface reconstruction, attributed to the occurrence of stripes running about 10° off the [001] direction. High-resolution STM images also identified different motifs at the surface for the different compounds (Fig. 2 ▸). Bipentagonal features are resolved at the Al_13_Co_4_(100) surface while pairs of bright features are observed at the Al_13_Fe_4_(010) surface, and fivefold motifs combined with pentagonal vacancies are visible at the Al_13_Ru_4_(010) surface (Ledieu *et al.*, 2017 ▸).

For Al_13_Fe_4_(010), the combination of theoretical calculations and experimental observations led to a model preserving the Henley-type clusters at the surface. The corresponding simulated STM images were in good agreement with the experimental images (Ledieu *et al.*, 2013 ▸). Further investigations based on dynamic LEED confirmed this result (Matilainen *et al.*, 2015 ▸). Several complementary techniques have been employed to reach a reliable model of the Al_13_Co_4_(100) surface. The combination of STM, calculations based on density functional theory (DFT) and LEED converged towards a surface terminating at puckered layers (hereafter P-layers, Fig. 1 ▸) where on average all Al atoms are present and protruding Co atoms are missing (Shin *et al.*, 2011 ▸; Fournée *et al.*, 2012 ▸). The surface structure showed heterogeneities, identified by STM, related to the partial occupancy of a few surface sites (Al ‘glue’ atoms, located in between Al bipentagonal motifs). The combination of surface X-ray diffraction and DFT pointed towards the same surface model, with partial occupancies for surface Co sites slightly buried in the P-type plane (Gaudry *et al.*, 2016 ▸).

Whether or not clusters are preserved at the surface must be linked to the strength of their intrinsic bonds and, as such, is expected to strongly depend on the atomic and electronic structures of the considered compounds. While the dimensionality of the chemical bonding network in o-Al_13_Co_4_ and Al_13_Fe_4_ has been investigated on the basis of electrical transport measurements (Dolinšek & Smontara, 2011 ▸), no theoretical extensive and systematic comparison of the compounds in the Al_13_TM_4_ (TM = Co, Fe, Ru, Rh) series has been carried out so far. Our work is based on a wide range of *ab initio* tools, based on DFT, in order to investigate the influence of the chemical composition on the pertinence of a bulk structure description based on clusters. Electronic structure calculations, including band structure calculations, projected density of states and projected crystal orbital Hamilton populations, highlight the different bonding characters of Al–Al, Al–TM and TM–TM pairs and how they contribute to the bulk cohesion. Methods based on infinitesimal displacements and harmonic approximations emphasize the impact of the chemical composition on phonon properties. Anisotropic thermal displacements are analysed and the singular behaviour induced by the cage structure is shown. Altogether, our results are used to discuss the relevance of the cage- versus layer-based descriptions. Finally, the interplay between the 3D bulk atomic arrangements and the 2D surface is discussed, based on surface energy calculations, before we present the conclusion.

## 2. Computational details and methods   

### 2.1. Bulk calculations   

The ground-state properties of the Al_13_TM_4_ structures, with TM = Fe, Co, Ru or Rh, are deduced from calculations based on DFT, using the plane-wave *Vienna* *ab initio* *simulation package* (*VASP*) (Kresse & Hafner, 1993 ▸, 1994 ▸, 1996*a* ▸,*b* ▸). The interaction between the valence electrons and the ionic core is described using the projector-augmented wave (PAW) method (Blöchl, 1994 ▸; Kresse & Joubert, 1999 ▸) within the generalized gradient approximation (GGA-PBE) (Perdew *et al.*, 1996 ▸, 1997 ▸), considering the valences for the atoms to be 3*s* ^2^3*p* ^1^ (Al), 4*s* ^1^3*d* ^7^ (Fe), 4*s* ^1^3*d* ^8^ (Co), 4*p* ^6^5*s* ^1^4*d* ^7^ (Ru) and 4*p* ^6^5*s* ^1^4*d* ^8^ (Rh). Spin polarization is not taken into account, as it was shown to be unnecessary for such Al-rich complex intermetallic compounds (Mihalkovič & Widom, 2007 ▸; Shin *et al.*, 2011 ▸). Total energies are minimized until the energy differences become less than 10^−5^ eV (respectively, 10^−8^ eV) between two electronic cycles during the structural optimizations (respectively, phonon calculations). Atomic structures are relaxed until the Hellmann–Feynman forces are as low as 0.02 eV Å^−1^. They are plotted using the *VESTA* software (Momma & Izumi, 2011 ▸).

Total energy calculations were performed using a cut-off energy (*E*_cut_) and a number of *k*-points within the Brillouin zone so as to achieve an energy accuracy better than 0.1 meV per atom (*E*_cut_ = 450 eV, Monkhorst–Pack *k*-points grid = 9 × 7 × 5 or equivalent). The reciprocal-space sampling was increased for electronic structure calculations (17 × 13 × 9 Monkhorst–Pack *k*-points grid) and the tetrahedron method with Blöchl corrections was used for Brillouin zone integrations. For the phonon calculations with supercells (2 × 1 × 1 or equivalent), we used a smaller *k*-point grid (2 × 2 × 2), in agreement with the setup of Mihalkovič & Widom (2007 ▸).

The phonon frequencies and the thermal displacements are determined using force constants derived from the calculation of the dynamic matrix based on the finite displacement method implemented in the *Phonopy* software (Togo & Tanaka, 2015 ▸). We used small atomic displacements (±0.01 Å). No imaginary mode was detected. The ellipsoid software was used to convert the thermal displacement parameters from the Cartesian to the crystal coordinate system (Deringer *et al.*, 2014 ▸; George *et al.*, 2015 ▸).

We used the projected crystal orbital Hamilton population (pCOHP) approach, implemented in the *LOBSTER* code (Dronskowski & Bloechl, 1993 ▸; Deringer *et al.*, 2011 ▸; Maintz *et al.*, 2013 ▸, 2016 ▸) to analyse the chemical bonding. This method re-extracts Hamilton-weighted populations from plane-wave electronic structure calculations to develop a tool analogous to the crystal orbital Hamilton population (COHP) method (Deringer *et al.*, 2011 ▸). The electron wavefunctions are projected onto the atomic local basis used for the DFT calculations: 3*s*3*p* for Al, 4*s*3*d* for Co and Fe, 4*p*5*s*4*d* for Ru and Rh. The charge spilling, *i.e.* electrons which cannot be projected onto the local basis, is found to be between 1 and 3% (1.09% for Al_13_Rh_4_ and 2.79% for Al_13_Fe_4_).

### 2.2. Surface energy calculations   

The surfaces have been modelled with seven-layer-thick symmetric slabs, separated by a void thickness (≃12 Å). Surface energies (γ_clean_) were computed as a function of the Al chemical potential: 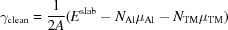
where *E*_slab_ is the total energy of the slab, μ_*i*_ and *N*_*i*_ the chemical potential and number of *i* species in the slab. The surface is considered to be in equilibrium with the underlying bulk, which constrains the chemical potentials in a range, *i.e.* 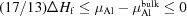 for Al, where Δ*H*_f_ is the formation energy of the complex phase.

Our values of the chemical potentials for the elemental Al, Fe, Co, Ru and Rh bulk crystals are in good agreement with experimental data: −3.52 eV for face-centred cubic Al (experimental: −3.39 eV), −4.86 eV for centred cubic Fe (experimental: −4.28 eV), −5.17 and −6.76 eV for hexagonal compact Co and Ru, respectively (experimental: −4.39 and −6.74 eV, respectively) (Kittel, 1996 ▸). The resulting formation energies for Al_13_TM_4_ compounds (TM = Fe, Co, Ru) are −0.329, −0.384, −0.522 eV per atom, respectively, in agreement with the values calculated by Mihalkovič & Widom (2004 ▸) (−0.349, −0.410, −0.548 eV per atom, respectively).

The Al_13_TM_4_ compounds have been identified as promising catalysts towards hydrogenation reactions (Armbrüster *et al.*, 2009 ▸, 2012 ▸; Piccolo, 2013 ▸; Piccolo & Kibis, 2015 ▸). Therefore, to investigate a possible modification of the surface structure during operating conditions (H_2_ atmosphere), the surface energies of the hydrogenated surfaces (γ_cover_) were evaluated by the sum of the clean surface energies (γ_clean_) and those with adsorbed species (γ_ads_) (Reuter *et al.*, 2002 ▸; Reuter & Scheffler, 2003 ▸; Posada-Pérez *et al.*, 2017 ▸): γ_cover_ = γ_clean_ + γ_ads_(*P*, *T*, 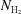) where *P*, *T* and 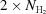 are the pressure, temperature and number of hydrogen atoms adsorbed on the surface. A simple thermodynamic model was used to compute γ_ads_:
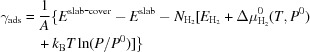
where 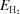 is the energy of H_2_ in vacuum, and 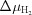 is the chemical potential of H_2_ calculated as 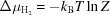 where *Z* is the partition function of the gas-phase H_2_ molecule and *k*_B_ is the Boltzmann constant. In the latter partition function, we only consider the translational and rotational contributions.

## 3. Bulk structures   

### 3.1. Atomic structures   

The Al_13_TM_4_ structures belong to the *C*2/*m* space group (102 and 51 atoms per cell for the conventional and unit cells, respectively). We also considered the Al_13_Co_4_ orthorhombic phase (o-Al_13_Co_4_), which crystallizes in the *Pmn*2_1_ space group, with 102 atoms per cell. These two structures share similarities. They are described as a stacking of flat (F) and puckered (P) layers along the pseudo-tenfold axis ([010] for monoclinic crystals, [100] for the orthorhombic one). As mentioned in Section 1, these structures are also described by a stacking of clusters. In the following, the term ‘cluster’ refers to the Henley-type cluster (Fig. 1 ▸), *i.e.* the pentagonal bipyramid.

The cell parameters deduced from the structural optimizations are gathered in Table 1 ▸. They are in good agreement with the experimental data. Here, full occupancies were considered in the theoretical approach, while partial occupancies are experimentally observed (Grin *et al.*, 1994*a* ▸,*b* ▸) and contribute to the stability of the compounds (Mihalkovič & Widom, 2007 ▸).

The relative differences 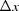 = 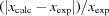 are smaller than 1% (respectively, 0.2%) for cell lengths (respectively, β angle). One exception is found for the Al_13_Rh_4_ compound (Δ*a* and Δ*b* are between 4 and 5%) (Chaudhury & Suryanarayana, 1983 ▸).

### 3.2. Phonon band structures   

**Figure 3:**
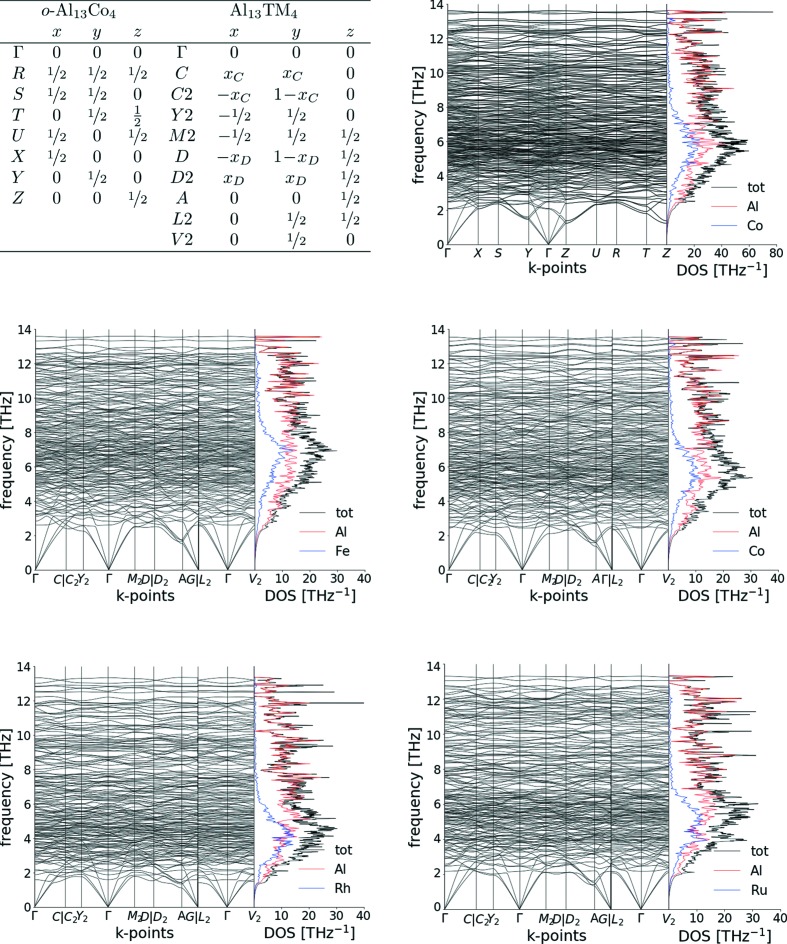
Phonon band structures and densities for o-Al_13_Co_4_ and monoclinic Al_13_TM_4_ compounds (TM = Co, Fe, Ru, Rh).

To evaluate the anisotropic displacement parameters, phonon calculations have been carried out. The resulting phonon band spectra are presented in Fig. 3 ▸ for o-Al_13_Co_4_ and monoclinic Al_13_TM_4_ (TM = Co, Fe, Ru, Rh). There are 153 and 306 branches in the phonon band structure, corresponding to the 3 × *N* degrees of freedom in the primitive unit cell for the monoclinic and ortho­rhombic structures, respectively. No band gap is observed, the optic modes arising from around 1.1–1.5 THz, in the A-point (respectively, Z-point) of the Brillouin zone for monoclinic (respectively, orthorhombic) structures. No clear difference between the averaged group velocities calculated perpendicularly or within the pseudo-tenfold axis is observed.

The phonon densities of states show a Debye behaviour at low energies and a maximum located around 4–6 THz. The position of the maximum is shifted to lower energies when moving from Al_13_Co_4_ and Al_13_Fe_4_ to Al_13_Ru_4_ and Al_13_Rh_4_, because 4*d* metals are heavier. For Al_13_Co_4_, our results for the vibrational density of states are in agreement with the experimentally measured ones (Mihalkovič *et al.*, 2000 ▸), and compare quite well with those reported by Mihalkovič & Widom (2007 ▸). However, in the latter case, the consideration of a single Al vacancy leads to a slight excess in the density of states at low frequency, which is not observed here because full occupancies were considered.

**Figure 4:**
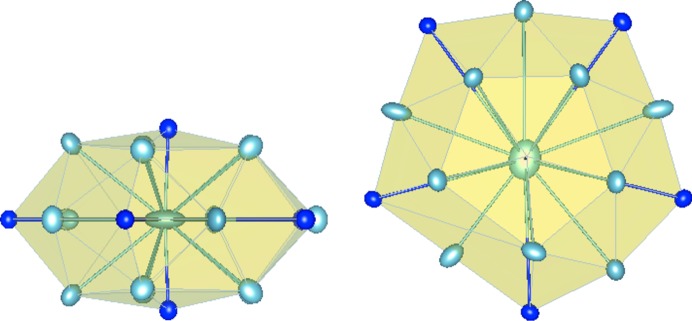
Top and side views of the anisotropic displacements for atoms located in the Henley-type cluster (Al_13_Co_4_ compound).

The previous phonon calculations were used to calculate the *U*_*ij*_ anisotropic thermal displacements. Larger anisotropic displacements are found for several Al atoms in the flat plane, the largest one being observed for the Al atom of the TM–Al–TM molecular group, with values for 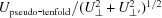 between 0.1 and 0.3 (Fig. 4 ▸). This is in agreement with the bonding picture, the Al atom located in the cluster centre being involved in strong bonds within the neighbouring Al_5_TM atomic arrangements located in the P-type plane above or below, while the covalent-like inter­actions with the surrounding atoms in the flat plane are found to be negligible (see Section 4).

### 3.3. Electronic band structures   

**Figure 5:**
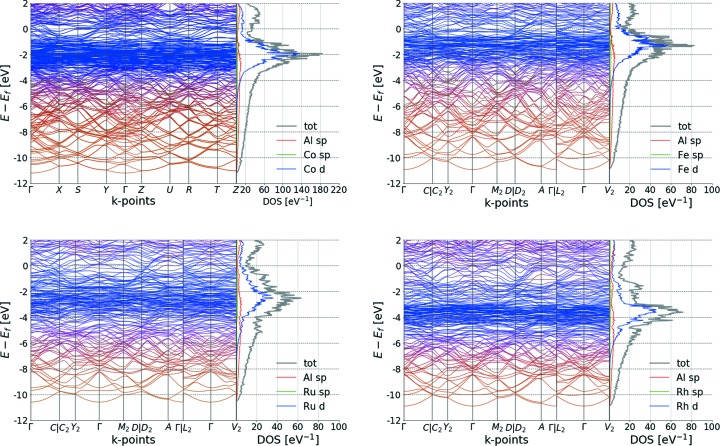
Band structures of the o-Al_13_Co_4_ and monoclinic Al_13_TM_4_ compounds (TM = Fe, Ru, Rh).

At low energy, the electronic structures of the Al_13_TM_4_ compounds (Fig. 5 ▸) present a parabolic dispersion, due to the free electron behaviour of the *sp*-like states. The orange colour of the bands shows the predominance of Al-*sp* states over TM-*sp* states. At higher energy, a strong maximum appears in the density of states (DOS), caused by localized and weakly dispersive TM-*d* states. This is in agreement with previous calculations (Mihalkovič & Widom, 2007 ▸; Manh *et al.*, 1995 ▸). The uniform colour of bands suggests a strong hybridization between Al-*sp* states and TM-*sp* states as well as between Al-*sp* states and TM-*d* states. A minimum in the DOS close to the Fermi energy (a pseudo-gap) is visible for all compounds, contributing to their stabilization (Mizutani & Sato, 2017 ▸; Trambly de Laissardière *et al.*, 2005 ▸). For Al_13_Ru_4_ the DOS at the Fermi energy is reduced to 35% compared with the value for Al_13_Fe_4_, in agreement with specific heat measurements (Wencka *et al.*, 2017 ▸) (Table 2 ▸).

The electron per atom ratio (e/a) using the atomic values recently computed by Mizutani *et al.* (Mizutani & Sato, 2017 ▸; Mizutani *et al.*, 2013 ▸; Mizutani, 2010 ▸) (1.00 for Rh, 1.03 for Co, 1.04 for Ru, 1.05 for Fe and 3.01 for Al) is 2.5 for the Al_13_TM_4_ compounds, *i.e.* slightly larger than the values usually observed for Hume-Rothery phases (Ferro & Saccone, 2008 ▸), which classifies them as polar intermetallics. The presence of the pseudo-gap may then be due to a combination of the Hume-Rothery stabilization mechanism with hybridization effects, as already highlighted for Al_9_Co_2_ (Trambly de Laissardière *et al.*, 2005 ▸).

## 4. Network of chemical bonds   

The bonding network in the Al_13_TM_4_ compounds is investigated in order to gain some insight into the various surface structures observed, the broken bond model being largely employed to account for surface energies (Ruvireta *et al.*, 2017 ▸).

### 4.1. Chemical bonding analysis   

**Figure 6:**
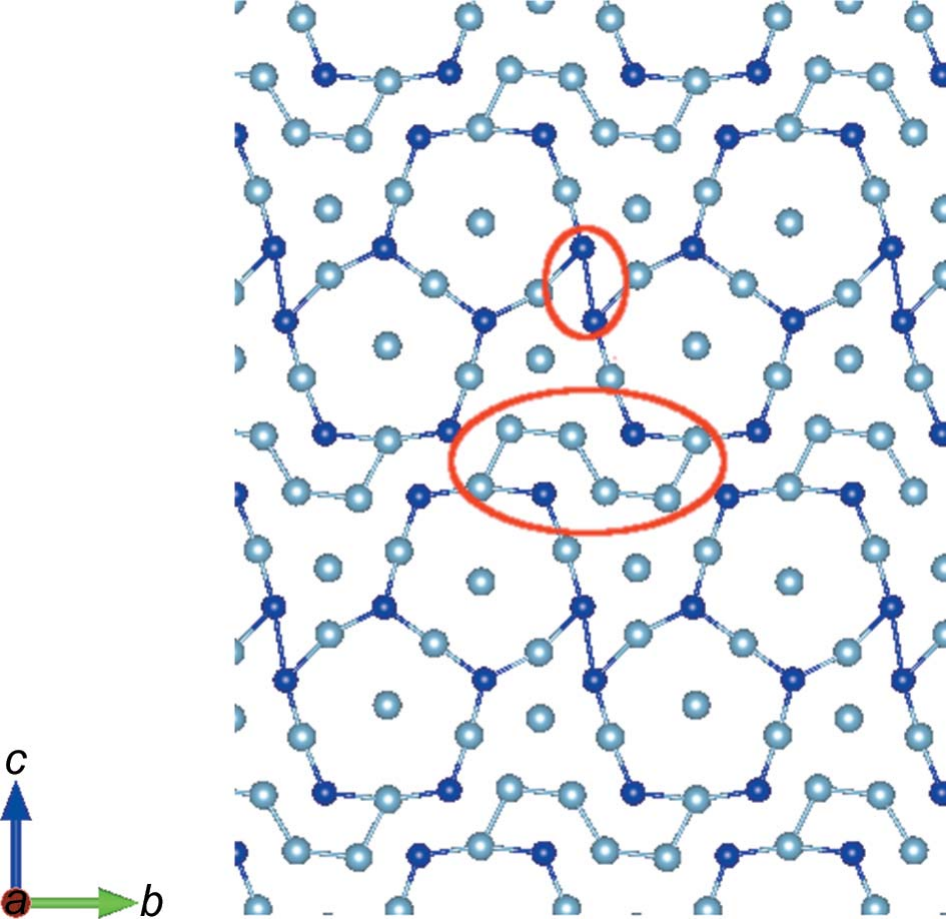
Structure of the o-Al_13_Co_4_ F-type plane highlighting the strongest Al–Al and Co–Co bonds (red circles), which are inter-cluster bonds, linking together bipentagonal atomic arrangements. Light blue = Al; dark blue = Co.

The chemical bond analysis based on the COHP curves and their integrated values (ICOHP, Table 3 ▸) revealed that the strongest bonds are homonuclear Al–Al bonds, located within the F-type atomic plane and ensuring the connection between clusters (Fig. 6 ▸). The strength of these bonds is rather high (larger than 2 eV per bond), despite quite large Al–Al distances (larger than 2.5 Å). This is consistent with the idea that the metallic bond is very closely related to the covalent (shared-electron-pair) bond, and that each atom in a metal may be considered as forming covalent bonds with neighbouring atoms, the covalent bonds resonating among the available interatomic positions (Pauling, 1947 ▸). The shortest Al–TM distances also lead to strong bonds. They are identified as those of the TM–Al–TM molecular group located inside the cluster, oriented parallel to the pseudo-tenfold axis, connecting the F- and P-type atomic planes, in agreement with the previous analysis by Grin *et al.* (Armbrüster *et al.*, 2011 ▸), and consistent with the NMR study by Jeglič *et al.* (2009 ▸, 2010 ▸). Very weak TM–TM bonds are found (≃0.2 eV per bond), with bonding distances close to 3 Å.

### 4.2. Cage- versus layer-based description   

To probe the stacked-layer structure of the Al_13_TM_4_ periodic decagonal approximants, we evaluate the P-type in-plane (*S*_in_^P-type^) and inter-plane (*S*_out_) bonding capacities by
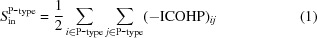


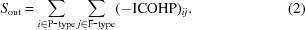
The ratio 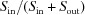 is calculated to be 24.5%, 26.8%, 24.4% and 28.4% for TM = Fe, Co, Ru, Rh, respectively. These results are consistent with the conclusions of Dolinšek & Smontara (2011 ▸), based on anisotropic resistivity measurements, stating that the stacked-layer description in terms of 2D atomic planes should only be regarded as a convenient geometrical approach to describe structures of the Al_13_TM_4_ quasicrystalline approximants, whereas their physical properties are those of true 3D solids.

**Figure 7:**
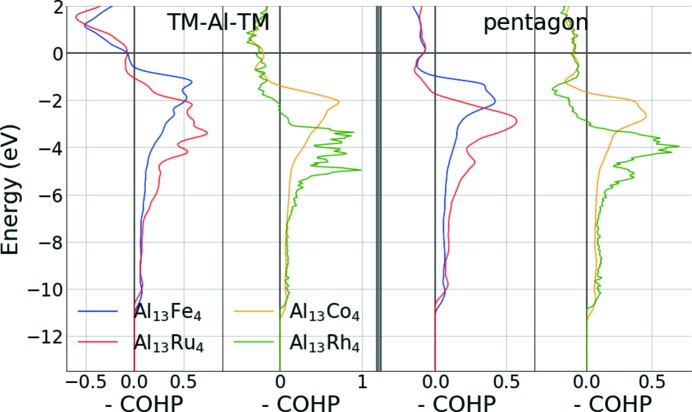
COHP curves for the TM–Al bonds of the TM–Al–TM molecular group, along with those showing the interactions of TM_P-type_ atoms with the surrounding Al atoms in the P-type plane (Al pentagonal arrangements).

Cluster stability is evaluated through the bonding capacity of TM atoms located in the P-type planes (TM_P-type_). Such atoms are bounded to three different types of Al neighbours: the Al atom within the TM–Al–TM molecular group, the surrounding Al atoms, located in the P-type plane (pentagonal arrangement), and the ones outside the cluster, within the flat plane (Fig. 1 ▸). Our results are presented in Table 4 ▸. For all compounds, the contributions to the bonding capabilities of the TM–Al–TM molecular group are around 16–17%. It is the largest for Al_13_TM_4_ with TM = Fe, Ru, because the corresponding states show almost no anti-bonding for TM = Fe, Ru (Fig. 7 ▸), while they are slightly anti-bonding for TM = Co, Rh. For all compounds, the intra-cluster Al–TM_P-type_ interactions contribute 69–70% to the bonding capabilities. More than 50% of these interactions are attributed to the closest Al pentagonal arrangement, even if slight anti-bonding TM–Al_P-type_ interactions at the Fermi level are revealed by the COHP curves. Intra-cluster contributions to the bonding capabilities of TM_P-type_ atoms are the strongest for Al_13_Fe_4_, and the lowest for o-Al_13_Co_4_ and Al_13_Rh_4_. An intermediate case is that of Al_13_Ru_4_, with Al–TM_P-type_ bonds within the TM_P-type_–Al–TM_P-type_ group as strong as those in Al_13_Fe_4_, while the strengths of TM–Al_F-type_ bonds are similar in Al_13_Ru_4_, o-Al_13_Co_4_ and Al_13_Rh_4_.

### 4.3. Bonding strengths and bonding distances   

**Figure 8:**
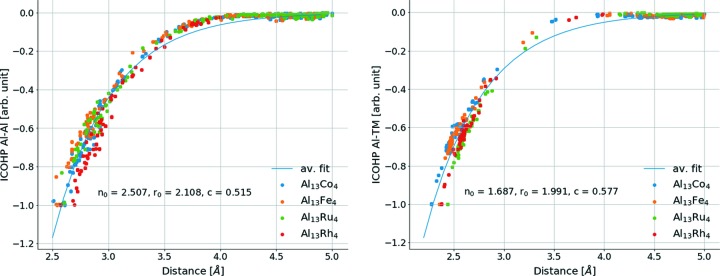
Bonding strength of Al–Al bonds in Al_13_TM_4_ compounds as a function of the distance. The fit uses the function 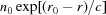.

Finally, when looking at the variation of bonding strength as a function of bonding distance (Fig. 8 ▸), an exponential behaviour is observed, in agreement with the exponential decrease in the bond number *n* with bonding distance *D*_*n*_ (in Å) initially proposed by Pauling (1947 ▸, 1960 ▸): 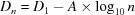 [equation (29) in Herman (1999 ▸)].

## 5. Bonding network and pseudo-tenfold surfaces   

From the bonding analysis, the P-type in-plane bonding capacities are evaluated to be in the range 20–30%. The clusters are found to be rather stable entities, the intra-cluster Al–TM_P-type_ interactions contributing 69–70% to the TM_P-type_ bonding capabilities. In the following, we discuss the consistency of these results with the pseudo-tenfold surface structures identified so far.

### 5.1. Clean surfaces   

**Figure 9:**
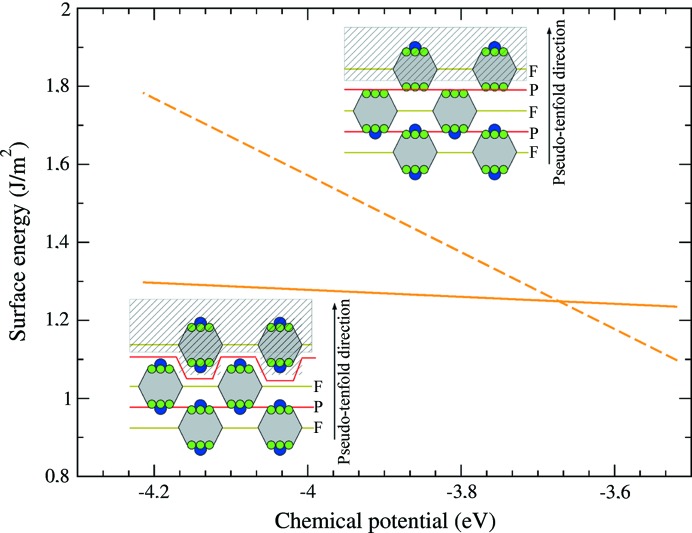
Surface energy of the A- (full line) and B-type (dashed line) models, calculated for o-Al_13_Co_4_(100).

Two surface models are considered in the following. The A-type model preserves the Henley-type clusters at the surface, while the B-type surface model terminates at P-layers where on average all Al atoms are present and protruding Co atoms are missing. The surface energies of these two models are plotted in Fig. 9 ▸. The A-type model, preserving the cluster structure at the surface, is found to be the most stable within a large domain of chemical potentials, for both Al_13_Co_4_(100) and Al_13_Fe_4_(010), in agreement with the bonding situation of the compounds. However, the surface structure observed for Al_13_Co_4_(100) is the B-type one, corresponding to the narrow range in the Al-rich region.

The surface structures of the Al_13_TM_4_ compounds with TM = Co and Fe reflect the duality between the description of the structure based (i) on a stacking of atomic planes (F- and P-type) perpendicular to the pseudo-tenfold direction, mirroring the periodic stacking of atomic planes with quasiperiodic in-plane atomic order found in decagonal quasicrystals, and (ii) on a stacking of Henley-type clusters. A dense Al-rich surface was identified for o-Al_13_Co_4_(100) and a highly corrugated surface, based on the preservation of the cluster structure at the surface, for m-Al_13_Fe_4_(010). They are consistent with the stronger character of intra-cluster bonds of m-Al_13_Fe_4_ compared with o-Al_13_Co_4_. They are also consistent with the slightly stronger in-plane bonding capacities of the Al_13_TM_4_ (TM = Fe, Co) P-type planes: 24.5% and 26.8% for m-Al_13_Fe_4_ and o-Al_13_Co_4_, respectively.

Compared with the related m-Al_13_Fe_4_(010) and o-Al_13_Co_4_(100) surfaces where superstructures are absent, the reconstruction observed for m-Al_13_Ru_4_(010) is thought to act as a strain relief mechanism. Here, the Ru atoms located in the P-type atomic plane are strongly bonded to the Al atom of the Ru–Al–Ru molecular group: 2.30 eV per bond, but a bonding capacity similar to that of m-Al_13_Fe_4_(010), *i.e.* 16.6%. However, the surface structures of m-Al_13_Fe_4_(010) and m-Al_13_Ru_4_(010) present quite large differences, since a reconstruction is observed in the case of m-Al_13_Ru_4_(010).

### 5.2. Surface under hydrogen atmosphere   

Both Al_13_Co_4_ and Al_13_Fe_4_ compounds have been identified as promising catalysts for hydrogenation reactions (Arm­brüster *et al.*, 2009 ▸, 2012 ▸; Piccolo, 2013 ▸; Piccolo & Kibis, 2015 ▸). The performances of the catalysts are attributed to the Al_5_TM atomic arrangements at the surface, in agreement with the site isolation concept. While such atomic arrangements have been experimentally observed for m-Al_13_Fe_4_(010), the surface structure determined so far under ultra-high vacuum for o-Al_13_Co_4_(100) consists of a dense Al-rich plane, leading to higher barriers for hydrogenation reactions (Krajčí & Hafner, 2011 ▸; Kandaskalov *et al.*, 2017 ▸).

Experimental conditions are known to have a strong effect on the surface structures. For example, slight deviations from an ordered alloy’s ideal stoichiometry in the subsurface or bulk region can drastically affect the surface composition (Ruban, 2002 ▸; Blum *et al.*, 2002 ▸). Such effects have been observed on o-Al_13_Co_4_(100) using two single crystals grown by two different techniques, which may present slightly different bulk compositions (within the stability range of the o-Al_13_Co_4_ phase) (Fournée *et al.*, 2012 ▸). However, in both cases, the surface is Al rich and no Co atoms were found to protrude at the surface.

**Figure 10:**
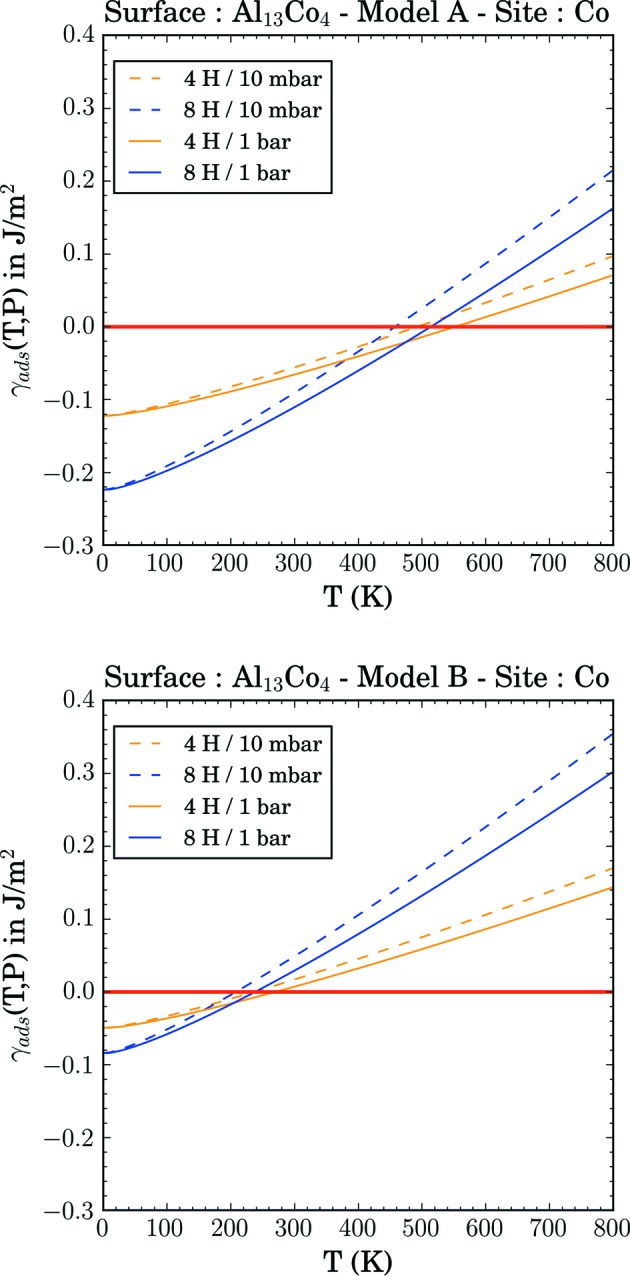
Modification of the o-Al_13_Co_4_(100) surface energy (γ_ads_) with atomic hydrogen adsorbates, for two different coverages, as a function of temperature and pressure.

When used as catalysts, the surface structure may evolve under operating conditions. In particular, exothermic adsorption on solid surfaces is known to reduce their surface energy (Mathur *et al.*, 2005 ▸). In the following, we evaluate the modification of the surface energy due to the adsorption of atomic hydrogen. We focus on o-Al_13_Co_4_(100) using the two previous surface models (A- and B-type models). We consider two different coverages (4 and 8 atomic H per surface cell, Fig. 10 ▸). Atomic hydrogen is adsorbed on the most favourable adsorption sites (Krajčí & Hafner, 2011 ▸; Kandaskalov *et al.*, 2014 ▸). The adsorption leads to a decrease in the surface energy, for temperatures below 400 and 200 K for the A- and B-type models, respectively. The stabilization is higher for the A-type model, because atomic hydrogen is more strongly adsorbed on the A-type model. The relative stabilization of the A-type model compared with the B-type one is found to be 0.07 and 0.14 J m^−2^ for the two considered coverages, respectively.

The surface energy difference calculated between the A- and B-type models is 0.14 J m^−2^ for μ_Al_ = μ_Al_^bulk^, *i.e.* larger or equal to the relative stabilization of the A-type model compared with the B-type one, when μ_Al_ = μ_Al_^bulk^, and for the considered adsorption configurations and coverages. For small atomic hydrogen coverages (four atoms per surface cell), our calculations, realized with the PBE approach, suggest that the considered surface structures are rather stable. However, larger atomic hydrogen coverages are likely to modify the surface structure.

## 6. Conclusion   

We reported a systematic investigation of the electronic structure, phonon properties and chemical bonding network of bulk Al_13_TM_4_ compounds (TM = Co, Fe, Ru, Rh). Electronic structure calculations highlight rather strong hybridization. The strong TM–Al–TM bond within the Henley-type cluster leads to a strong anisotropy in the thermal displacement of the central Al atom. From the bonding analysis, the clusters are found to be rather stable entities, the intra-cluster Al–TM_P-type_ interactions contributing 69–70% to the TM_P-type_ bonding capabilities.

Structural differences between the o-Al_13_Co_4_(100) and m-Al_13_Fe_4_(010) surfaces have been observed for several different samples, different growth modes and for quite different annealing temperatures: bipentagonal motifs are systematically observed for o-Al_13_Co_4_(100), whereas they are never resolved in the case of m-Al_13_Fe_4_(010). The calculated strengths of the Al–TM bonds within the TM–Al–TM mol­ecular groups provide an understanding of the different surface structures observed. Since intra-cluster bonds are stronger for m-Al_13_Fe_4_ compared with o-Al_13_Co_4_, and in-plane bonding capacities of the P-type planes are stronger for o-Al_13_Co_4_ compared with m-Al_13_Fe_4_, Henley-type clusters are preserved at the m-Al_13_Fe_4_(010) surface while they are truncated at the o-Al_13_Co_4_(100) surface.

The possible interactions with adsorbates lead to a decrease in the calculated surface energies, at low temperatures (*T* < 400–500 K and *T* < 200–300 K for the A- and B-type models, respectively). The stabilization depends on the chemical potentials of the adsorbate, as well as on those of the Al and TM atoms. This highlights the importance of *operando* conditions when considering applications for these surfaces.

Nevertheless, the specific atomic arrangements at the surface induced by the intrinsic cluster substructure of complex intermetallic compounds affect the surface properties. Several adsorption studies highlight the role of specific sites resulting from the cut by the surface plane through the cluster units identified in the bulk solid (Unal *et al.*, 2009 ▸; Fournée *et al.*, 2014 ▸; Ledieu *et al.*, 2009 ▸; Krajčí & Hafner, 2008 ▸). On fivefold Al-based quasicrystalline surfaces, it leads to the two famous ‘dark stars’ and ‘white flowers’ sites (Unal *et al.*, 2009 ▸), which are identified as favourable atomic and molecular adsorption sites (Cai *et al.*, 2003 ▸; McGrath *et al.*, 2002 ▸). On approximant and related phases, signatures of the cluster substructure at the surface also lead to specific chemically active sites. For example, a possible reaction path for the semi-hydrogenation of acetylene on o-Al_13_Co_4_(100), identified as a performant catalyst for this reaction (Armbrüster *et al.*, 2009 ▸, 2012 ▸), is predicted to involve the protruding clusters (CoAl_5_ ensemble) (Krajčí & Hafner, 2011 ▸). Given the structural variety of complex intermetallics, described by stackings of very diversified clusters naturally present in the bulk structure, we can hope to control the surface properties by the selection of clusters that emerge at the surface as active centres, giving rise to multiple applications at the nanoscale.

## Figures and Tables

**Table 1:** Cell parameters resulting from structural optimization, for Al_13_TM_4_ monoclinic structures (*C*2/*m* space group) with TM = Fe, Co, Ru, Rh The case of the orthorhombic structure for Al_13_Co_4_ (*Pmn*2_1_ space group) is considered as well.

		*a* (Å)	*b* (Å)	*c* (Å)	β (°)
Al_13_Fe_4_	Calculated	15.43	8.03	12.43	107.70
	Experimental (Grin *et al.*, 1994*b* ▸)	15.492	8.078	12.471	107.69
	Experimental (Kazumasa *et al.*, 2012 ▸)	15.495	8.089	12.485	107.70
Al_13_Co_4_	Calculated	15.14	8.19	12.40	107.73
	Experimental (Hudd & Taylor, 1962 ▸)	15.183	8.122	12.340	107.81
o-Al_13_Co_4_	Calculated	8.195	12.40	14.43	90
	Experimental (Grin *et al.*, 1994*a* ▸)	8.158	12.342	14.452	90
Al_13_Ru_4_	Calculated	15.94	8.30	12.82	107.76
	Experimental (Edshammar, 1965 ▸)	15.862	8.188	12.736	107.77
	Experimental (Murao *et al.*, 2011 ▸)	15.860	8.192	12.742	107.77
Al_13_Rh_4_	Calculated	15.64	8.45	12.79	107.92
	Experimental (Chaudhury & Suryanarayana, 1983 ▸)	16.36	8.05	12.79	107.77

**Table 2:** Position of the *d*-band maximum and density of states at the Fermi energy for the considered compounds

	Al_13_Fe_4_	o-Al_13_Co_4_	Al_13_Ru_4_	Al_13_Rh_4_
max(*d*-TM) (eV)	−1.29	−1.98	−2.50	−3.49
*n*(*E*_F_) (states per atom per eV)	0.286	0.316	0.179	0.254
			0.219 (Manh *et al.*, 1995 ▸)	

**Table 3:** Average ICOHP/bond values (eV per bond), along with the corresponding average distance (in parentheses, in Å), for the strongest Al–Al, TM–TM and Al–TM bonds in Al_13_TM_4_ compounds

	Al–Al	Al–TM	TM–TM
Al_13_Fe_4_	2.60	1.83	0.23
	(2.54 Å)	(2.35 Å)	(2.91 Å)
o-Al_13_Co_4_	2.46	1.62	0.18
	(2.61 Å)	(2.27 Å)	(2.89 Å)
Al_13_Ru_4_	2.71	2.29	0.40
	(2.57 Å)	(2.44 Å)	(3.04 Å)
Al_13_Rh_4_	2.40	2.01	0.18
	(2.57 Å)	(2.38 Å)	(3.02 Å)

**Table 4:** Average ICOHP/bond values (eV per bond) and percentage contributions (the contributions of the bonding relative to a given atom are evaluated by the sum of the ICOHP/bond values of the nearest neighbouring interactions weighted by the respective bond frequencies) of the respective interactions to the net bonding capabilities around the TM atoms located in the P-type atomic plane Three types of Al neighbours are considered: the Al atoms within the TM–Al–TM molecular group, the surrounding Al atoms in the P-type plane (pentagonal arrangement), and those outside the cluster, in the F-type plane (Fig. 1 ▸).

	TM–Al (TM–Al–TM)		TM–Al_F-type_		TM–Al_P-type_	
	eV per bond	%	eV per bond	%	eV per bond	%
Al_13_Fe_4_	1.83	16.8	1.14	31.3	1.13	51.9
o-Al_13_Co_4_	1.62	16.0	1.03	30.6	1.08	53.3
Al_13_Ru_4_	2.30	16.6	1.41	30.5	1.47	52.3
Al_13_Rh_4_	2.01	16.1	1.27	30.4	1.34	53.6
